# Daylight Savings Time and Acute Myocardial Infarction

**DOI:** 10.1001/jamanetworkopen.2025.30442

**Published:** 2025-09-09

**Authors:** Jennifer A. Rymer, Shuang Li, Karen Chiswell, Aman Kansal, Michael G. Nanna, Jorge Antonio Gutierrez, Dmitriy N. Feldman, Sunil V. Rao, Rajesh V. Swaminathan

**Affiliations:** 1 Division of Cardiology, Duke University Hospital, Durham, North Carolina; 2 Duke Clinical Research Institute, Durham, North Carolina; 3 Yale School of Medicine, New Haven, Connecticut; 4 Weill Cornell Medical College, NewYork-Presbyterian Hospital, New York; 5 New York University Grossman School of Medicine and New York University Langone Health System, New York

## Abstract

**Importance:**

Previous data suggest that the time changes associated with daylight savings time (DST) may be associated with an increased incidence of acute myocardial infarction (AMI).

**Objective:**

To determine whether the incidence of patients presenting with AMI is greater during the weeks during or after DST and compare the in-hospital clinical events between the week before DST and after DST.

**Design, Setting, and Participants:**

This cross-sectional study examined patients enrolled in the Chest Pain MI Registry from 2013 to 2022. The study included patients who presented 1 week before DST, during the week of DST, or 1 week after DST (spring or fall) and assessed incidence of AMI, in-hospital mortality, stroke, and clinical outcomes during those times and compared the DST weeks with the week before or after. Data were analyzed from March 2024 to May 2025.

**Main Outcome and Measure:**

The main outcome was in-hospital mortality rate. The incidence ratio (IR) of AMI cases was calculated using the observed number of patients with AMI in the DST week divided by the number of patients with AMI who arrived 1 week before or after DST.

**Results:**

The final cohort included 168 870 patients (median [IQR] age, 65 [56-75] years, 57 023 females [33.8%]; 111 847 males [66.2%]) at 1124 hospitals during the study period. There were 28 678 patients (17.0%) with AMI treated during spring DST, 28 596 (16.9%) the week before, and 28 169 (16.7%) the week after. There were 27 942 patients (16.5%) with AMI treated during fall DST, 27 365 (16.2%) the week before, and 28 120 (16.7%) the week after. Patient characteristics were similar for the spring and fall DST analyses (spring and fall DST median [IQR] age across groups: 65 [56-74] years and 65 [56-75] years, respectively), and there were 28 725 females (33.6%) in the spring and 28 298 females (33.9%) in the fall. There was no significant difference in the incidence of AMI for the following time periods: spring DST week vs 1 week prior, spring DST week vs 1 week after, fall DST week vs 1 week prior, and fall DST week vs 1 week after. There were no significant differences in adjusted in-hospital outcomes for the 1 week before or after fall or spring DST.

**Conclusions and Relevance:**

This study found no significant difference in the incidence rates of AMI in DST weeks compared with the week before or after. Additionally, there were no differences in in-hospital clinical outcomes.

## Key Points

**Question:**

What is the association of daylights savings time (DST) with the incidence and outcomes of myocardial infarction?

**Findings:**

In this cross-sectional study of 168 870 patients, no significant differences were found in the incidence rates of acute myocardial infarction in DST weeks compared with the week before or after. Additionally, there were no significant differences in outcomes, including in-hospital mortality and stroke, between these weeks.

**Meaning:**

These findings suggest that that there is no significant association between DST and the incidence and outcomes of patients presenting with myocardial infarction.

## Teaser

This cross-sectional study examines the incidence of patients presenting with myocardial infarction in the weeks before, during, and after the start and end of daylight savings time.

## Introduction

Since 1918, daylight savings time (DST) is the time each year when clocks are advanced 1 hour in the US. This change creates an additional hour of sunlit time during the summer. When returning to standard time, clocks are moved back 1 hour, thus moving an hour of sunlit time to the morning (ie, spring forward, fall back). DST begins at 2 am on the second Sunday of March and ends at 2 am on the first Sunday of November.

There are older studies examining the incidence of acute myocardial infarction (AMI) during and after DST. However, the pharmacologic and invasive treatments for AMI have evolved, so that the contemporary association between DST and the incidence of AMI is unclear. Using data from the Swedish registry, investigators found that the incidence of AMI was associated with a significant increase during the first 3 days after transition to DST in the spring.^1^ The incidence ratio (IR) for AMI for the first week after the spring shift was significantly increased, 1.051 (95% CI, 1.032 to 1.071). In contrast, the IR for AMI for the week after transitioning out of DST was not increased. In another analysis performed over a decade ago, Jiddou et al^2^ examined data from several Michigan hospitals to determine the association of DST on incidence of AMI. They found a significantly greater incidence of non–ST-segment AMI after the transition to DST (*P* = .02). Finally, another analysis using data from the BMC2 database demonstrated a 24% increase in AMI incidence on the Monday after spring DST.^3^

As much of the data examining the association of DST and AMI incidence is over a decade old and from limited registry datasets, we sought to examine the incidence of AMI in the weeks before, during, and after DST and return to standard time using the National Cardiovascular Data Registry (NCDR) Chest-Pain MI Registry.^4^ Furthermore, we aimed to examine the association between DST and adverse clinical events during the weeks before, during, and after time changes.

## Methods

### Data Source

Data for this cohort study were obtained from the National Cardiovascular Data Registry (NCDR) Chest Pain–MI Registry, a voluntary registry of 1323 US sites that collects data on consecutive patients admitted with AMI at participating centers across the US. Patient characteristics, including medical history, clinical presentation details, in-hospital treatments, and in-hospital outcomes, are abstracted by trained abstractors at participating sites via medical record review. Regular audits and data quality checks are performed to ensure data accuracy within the registry. The Duke University Health System institutional review board approved the study with a waiver of informed consent because data were from a national retrospective registry. This report follows the Strengthening the Reporting of Observational Studies in Epidemiology (STROBE) reporting guideline for cross-sectional studies.

### Study Cohort

The study population included unique, consecutive patients included in the Chest Pain–MI Registry between January 1, 2013, and December 31, 2022. The study cohort population is described in the results (eFigure 1 in Supplement 1).

### Study Definitions

#### Daylight Savings Time

In the US, since 2007, DST has started on the second Sunday in March and ended on the first Sunday in November with time changes taking place at 2:00 am local time. eTable 1 in Supplement 1 includes the dates of spring DST and the end of DST in the fall (standard time) during the study period, including the dates of the week before and after DST. For the purposes of this analysis the beginning of DST was termed spring DST and the end of DST in the fall was term fall DST.

#### Outcomes

We examined in-hospital mortality (primary outcome), in-hospital stroke, revascularization for non-ST elevation myocardial infarction (NSTEMI), and reperfusion for STEMI. For in-hospital stroke, we defined this outcome as any cerebrovascular accident or stroke (if from version 2 of the data collection form) or as any hemorrhagic, ischemic, or undetermined stroke (if from version 3 of the data collection form). Revascularization for NSTEMI included percutaneous coronary intervention (PCI) or coronary artery bypass grafting (CABG). Reperfusion for STEMI included administration of lytics or performance of primary PCI.

### Statistical Analysis

We examined the change in incidence of AMI for the week of spring DST and fall DST when compared with the incidence of AMI in the week before and after each of the DST weeks. The incidence ratio (IR) of AMI cases was calculated using the observed number of patients with AMI in the DST week divided by the number of patients with AMI arrived 1 week before or after DST. On the first day of DST (because of spring forward), there is only 23 hours, so the number of patients with AMI on this day was calculated by the observed number of patients with AMI divided by 23 and multiplied by 24 hours. Because of fall back, the day of fall DST has 25 hours, so the number of patients with AMI on the first day of fall DST was calculated by the observed number of patients with AMI divided by 25 and multiplied by 24 hours. The mean IR and a 95% CI of overall years will be calculated using the IR estimates from each year of the study (n = 10 years), and assuming the yearly estimates are independent. We performed similar calculations for each day of the DST week and compared with the same day of the week before and after DST (ie, the Sunday of DST compared with the Sunday of the week before and after DST). We also presented the mean IR and 95% CI for each year during the study period. We examined these associations for NSTEMI and STEMI.

We examined the baseline demographic and clinical characteristics of patients treated for AMI during the following time periods: (1) 1 week before spring DST, (2) spring DST week, (3) 1 week after spring DST week, (4) 1 week before fall DST, (5) fall DST week, and (6) 1 week after fall DST week. For spring and fall DST, groups 1 to 3 and 4 to 6, respectively, were compared. Race and ethnicity of patients was based on a a self-reported assessment. The Kruskal-Wallis test was used to compare continuous variables. The χ^2^ test was used for the comparison of categorical variables.

We summarized the observed number and percentages of patients with in-hospital events between the DST weeks and weeks either before or after the DST weeks. We performed multivariable logistic regression models to examine the association of DST with the odds of in-hospital events. We used generalized estimating equations (GEE) logistic regression models to account for clustering (ie, statistical dependence) of observations from the same hospital to compare event occurrence between comparison groups of patients after adjusting for patient covariates. The GEE method was implemented with a compound symmetric working correlation matrix and empirical (sandwich) standard error estimates. Covariates for risk adjustment are included in eTable 2 in Supplement 1. Continuous covariates, such as age, systolic blood pressure, heart rate, body mass index (BMI), left ventricular ejection fraction value, initial estimated glomerular filtration rate, and initial troponin ratio (upper level of normal), were fitted with a restricted cubic spline transformation with 3 knots at 10%, 50%, and 90% of its distribution of the subpopulation in each model. Since the percentage of missingness was small (less than 5%) for all the covariates in the models, missing continuous variables of patient characteristics were imputed to the STEMI and sex-specific median of the nonmissing values, and missing categorical variables of patient characteristics were imputed to the mode.

Lastly, we performed sensitivity analyses examining the IR for AMI, comparing the incidence during the DST weeks with the weeks before or after the DST week in the states of Hawaii and Arizona, where DST does not exist. We also compared the IR for AMI for DST weeks vs 3 weeks before or 3 weeks after the DST week for the full study period. This allowed us to ensure that there was not a delay in change in incidence after 1 week before or after DST weeks.

Hypothesis testing was 2-sided, and a *P* < .05 was considered significant for all analyses unless otherwise stated. All statistical analyses were performed using SAS version 9.4 software (SAS Institute). Data were analyzed from MArch 2024 to May 2025.

## Results

There are 2 498 903 patients enrolled in the Chest Pain-MI Registry from 1323 hospitals. We excluded 499 681 patients enrolled in the registry from years outside of the study time period, 342 412 patients for which only a short form data collection form is available; and 181 587 patients not admitted for a STEMI or NSTEMI. Additionally, we excluded 1 304 433 patients who did not present with an AMI in the week prior to, the weeks during the transition to and away from daylight savings time (DST), or the week after transition to or from DST in the spring or fall. Finally, we also excluded 1920 patients living in Arizona or Hawaii as these states do not participate in DST. The final study population used in the current study included 168 870 patients at 1124 hospitals between 2013 to 2022.

The median (IQR) age was 65 (56-75) years and 57 023 were females (33.8%). There were 28 678 patients with AMI treated during spring DST (17.0%), 28 596 the week before (16.9%), and 28 169 the week after (16.7%). There were 27 942 patients with AMI treated during fall DST (16.5%), 27 365 the week before (16.2%), and 28 120 the week after (16.7%).

Table 1 describes the baseline demographic and clinical characteristics for patients treated with AMI on the week before spring DST, during the week of spring DST, and 1 week after spring DST. There were no significant differences in patient characteristics in terms of age, sex, race, or clinical characteristics between the 3 weeks examined (Table 1). Similarly, Table 2 describes the baseline demographic and clinical characteristics for patients treated with AMI on the week before fall DST, during the week of fall DST, and 1 week after fall DST. There were no significant differences in demographic or clinical characteristics between patients presenting with AMI on these various weeks. Patient characteristics were similar for the spring and fall DST analyses (spring and fall DST median [IQR] age across groups: 65 [56-74] years and 65 [56-75] years, respectively), and there were 28 725 females (33.6%) in the spring and 28 298 females (33.9%) in the fall.

**Table 1.** Baseline Demographic and Clinical Characteristics of Patients Presenting With Acute Myocardial Infarction (AMI) Before, During, and After Spring DST During the Study Period

Covariate	Patients, No. (%)			*P* value
	One week before spring DST week (n = 28 596)	Spring DST week (n = 28 678)	One week after spring DST week (n = 28 169)	
Demographics				
Age, No.	28 596	28 678	28 169	.07
Median (IQR)	65 (56-74)	65 (56-75)	65 (55-74)	NA
Sex				
Female	9663 (33.8)	9593 (33.4)	9469 (33.6)	.69
Male	18 933 (66.2)	19 085 (66.6)	18 700 (66.4)	
BMI	27 577	27 625	27 161	.12
Median (IQR)	28.9 (25.4-33.3)	28.8 (25.4-33.2)	28.8 (25.3-33.2)	
Race				
Asian	635 (2.2)	624 (2.2)	560 (2.0)	.39
Black	3244 (11.3)	3158 (11.0)	3084 (11.0)	
White	24 020 (84.0)	24 169 (84.3)	23 857 (84.7)	
Cardiac status				
STEMI	10 706 (37.4)	10 752 (37.5)	10 629 (37.7)	.74
Heart failure	3665 (12.8)	3695 (12.9)	3600 (12.8)	.92
Cardiogenic shock	1107 (3.9)	1079 (3.8)	1070 (3.8)	.79
Cardiac arrest	1036 (3.6)	1027 (3.6)	984 (3.5)	.69
Medical history				
Hypertension	21 473 (75.1)	21 722 (75.7)	21 130 (75.0)	.08
Dyslipidemia	17 190 (60.1)	17 384 (60.6)	16 894 (60.0)	.14
Dialysis	740 (2.6)	752 (2.6)	724 (2.6)	.93
Diabetes	10 236 (35.8)	10 347 (36.1)	9916 (35.2)	.09
Prior AMI	6583 (23.0)	6459 (22.5)	6371 (22.6)	.37
Heart failure	3559 (12.5)	3684 (12.9)	3539 (12.6)	.32
Prior PCI	7143 (25.0)	7087 (24.7)	7014 (24.9)	.84
Prior CABG	3485 (12.2)	3352 (11.7)	3386 (12.0)	.21
Atrial fibrillation/flutter	2555 (8.9)	2669 (9.3)	2558 (9.1)	.29
Peripheral artery disease	2361 (8.3)	2431 (8.5)	2358 (8.4)	.63
Procedure information				
STEMI	10 706 (37.4)	10 752 (37.5)	10 629 (37.7)	.74
Coronary angiography	25 580 (91.9)	25 554 (91.6)	25 198 (91.7)	.44
Primary PCI for STEMI (among patients without missing PCI data)	7507 (91.4)	7506 (91.6)	7426 (91.3)	.78
Door to balloon time, median (IQR), min	57 (42-74)	57 (42-75)	58 (43-74)	.36
Revascularization for NSTEMI	11 146 (62.3)	11 004 (61.4)	10 814 (61.7)	.20
Thrombolytics	573 (6.2)	588 (6.3)	585 (6.4)	.84
Coronary artery bypass grafting for NSTEMI or STEMI	2274 (8.0)	2297 (8.0)	2276 (8.1)	.85
LVEF, No.	26 842	26, 824	26 418	.64
LVEF, Median (IQR), %	52 (40-59)	53 (40-60)	53 (40-59)	
Hospital covariates				
Total No. of beds	373 (273-552)	366 (237-546)	367 (237-551)	.51
Hospital type				
Urban	15 605 (54.6)	15 549 (54.2)	15 267 (54.2)	.81
Hospital region				
Midwest	7424 (26.0)	7514 (26.2)	7355 (26.1)	.95
Northeast	2529 (8.8)	2543 (8.9)	2488 (8.8)	
South	14 568 (51.0)	14 537 (50.7)	14 388 (51.1)	
West	4062 (14.2)	4077 (14.2)	3931 (14.0)	
Other covariate				
Arrival year after 2020	7019 (24.6)	6989 (24.4)	6547 (23.2)	<.001

Abbreviations: BMI, body mass index (calculated as weight in kilograms divided by height in meters squared); CABG, coronary artery bypass grafting; DST, daylight savings time; LVEF, left ventricular ejection fraction; NA, not applicable; NSTEMI, non–ST-elevation myocardial infarction; PCI, percutaneous coronary intervention; STEMI, ST-elevation myocardial infarction.

**Table 2.** Baseline Demographic and Clinical Characteristics of Patients Presenting With Acute Myocardial Infarction (AMI) Before, During, and After Fall Daylight Savings Time (DST) During the Study Period

Covariate	Patients, No. (%)			*P* value
	One week before fall DST end week (n = 27 365)	Fall DST end week (n = 27 942)	One week after fall DST end week (n = 28 120)	
Demographics				
Age, No.	27 365	27 942	28 120	NA
Age, median (IQR)	65 (56-75)	65 (56-74)	65 (55-75)	.50
Female	9294 (34.0)	9349 (33.5)	9655 (34.3)	.09
Male	XX	XX	XX	
BMI, No.	26 371	26 884	27 056	NA
BMI, median (IQR)	28.7 (25.2-33.1)	28.9 (25.3-33.2)	28.9 (25.3-33.2)	.27
Race				
Asian	596 (2.2)	602 (2.2)	637 (2.3)	.30
Black	3028 (11.1)	3077 (11.0)	3103 (11.0)	
White	23 076 (84.3)	23 558 (84.3)	23 708 (84.3)	
Cardiac status				
STEMI	10 251 (37.5)	10 580 (37.9)	10 537 (37.5)	.53
Heart failure	3470 (12.7)	3485 (12.5)	3569 (12.7)	.68
Cardiogenic shock	1011 (3.7)	1097 (3.9)	1034 (3.7)	.23
Cardiac arrest	931 (3.4)	1025 (3.7)	977 (3.5)	.21
Medical history				
Hypertension	20 651 (75.5)	21 114, (75.6)	21 236, (75.5)	.97
Dyslipidemia	16 499 (60.3)	16 967 (60.7)	17 085 (60.8)	.35
Dialysis	711 (2.6)	780 (2.8)	732 (2.6)	.28
Diabetes	9903 (36.2)	10 171 (36.4)	10 296 (36.6)	.58
Prior AMI	6203 (22.7)	6309 (22.6)	6417 (22.8)	.80
Heart failure	3487 (12.7)	3552 (12.7%)	3590 (12.8)	.98
Prior PCI	6841 (25.0)	7124 (25.5)	7091 (25.2)	.36
Prior CABG	3114 (11.4)	3340 (12.0)	3223 (11.5)	.07
Atrial fibrillation/flutter	2519 (9.2)	2562 (9.2)	2630 (9.4)	.69
Peripheral artery disease	2157 (7.9)	2224 (8.0)	2271 (8.1)	.69
Procedure information				
STEMI	10 251 (37.5)	10 580 (37.9)	10 537 (37.5)	.54
Coronary angiography	24 530 (92.0)	25 033 (92.1)	25 186 (92.2)	.65
Primary PCI for STEMI (among patients without missing PCI data)	7101 (81.1)	7273 (81.4)	7134 (80.8)	.81
Door to balloon time, median, (IQR), min	58 (42-74)	58 (43-74)	58 (43-75)	.35
Revascularization for NSTEMI	10 720 (62.6)	10 764 (62.0)	10 985 (62.5)	.45
Thrombolytics	522 (6.0)	572 (6.4)	596 (6.8)	.11
Coronary artery bypass grafting for NSTEMI or STEMI	2161 (7.9)	2162 (7.7)	2242 (8.0)	.58
LVEF, No.	25 665	26 151	26 361	
LVEF, Median (IQR), %	53 (40-58)	53 (40-60)	53 (40-58)	.55
Hospital covariates				
Total No. of beds	367 (237-550)	364 (235-546)	364 (235-546)	.30
Hospital type				
Urban	14 819 (54.2)	15 070 (54.0)	15 108 (53.7)	.56
Hospital region				
Midwest	7187 (26.3)	7475 (26.8)	7434 (26.4)	.42
Northeast	2386 (8.7)	2458 (8.8)	2402 (8.5)	
South	13 958 (51.0)	14 046 (50.3)	14 215 (50.6)	
West	3824 (14.0)	3951 (14.1)	4059 (14.4)	
Other covariate				
Arrival year after 2020	6518 (23.8)	6627 (23.7)	6819 (24.3)	.29

Abbreviations: BMI, body mass index (calculated as weight in kilograms divided by height in meters squared); CABG, coronary artery bypass grafting; LVEF, left ventricular ejection fraction; NA, not applicable; NSTEMI, non–ST-elevation myocardial infarction; PCI, percutaneous coronary intervention; STEMI, ST-elevation myocardial infarction.

eTable 3 in Supplement 1 describes the incidence ratios (IRs) for DST weeks vs the week before or after the DST week for the full study period. There was no significant difference in the observed incidence of AMI in any DST week compared with the weeks before or after for both fall and spring DST for the full study period. The Figure shows the IR of AMI presentations for spring DST and fall DST compared with the weeks before and after for each year during the study period. There was a 21% increase (incidence ratio, 1.21) in the incidence of patients presenting with AMI during the week of spring DST in 2020 compared with the week after; there was a 6% decrease (incidence ratio 0.94) in the incidence of AMI during the week of spring DST in 2020 compared with the week before. eFigures 2A and 2B in Supplement 1 depict the IR of AMI presentations for each day of the week of spring DST and fall DST vs the week prior, and vs the week after for the entire study period.

**Figure.**
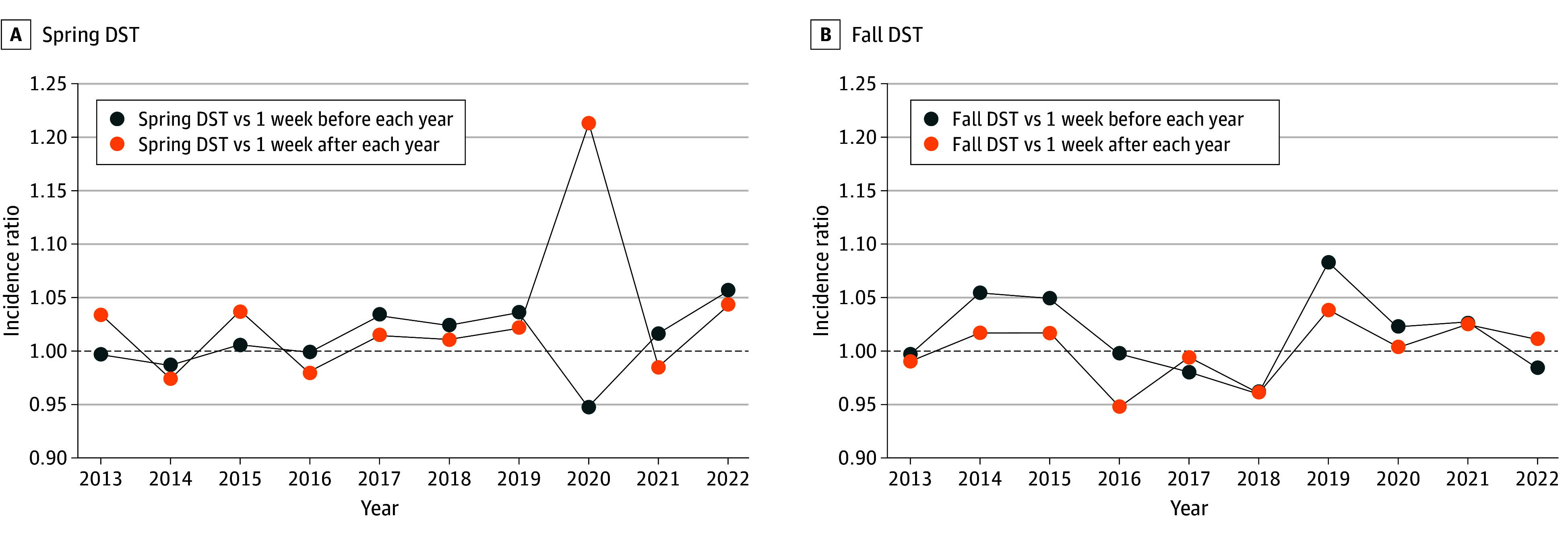
Incidence Ratio (IR) of Myocardial Infarction Presentations on the Week of Spring Daylight Savings Time (DST) and Fall DST Compared With the Weeks Before and After for Each Year During the Study Period There was no significant difference in the observed incidence of acute myocardial infarction in any DST week compared with the weeks before or after for both fall and spring DST for the full study period.

eTables 4 and 5 in Supplement 1 show that the number and percentage of patients in the weeks before, during, and after DST who had an in-hospital clinical event. The rates of in-hospital death were 4.5% (1223 of 27 152 patients) in the week before spring DST, 4.6% (1245 of 27 235 patients) in the week of spring DST, and 4.4% (1193 of 26 2753 patients) in the week after spring DST. The rates of in-hospital death were 4.8% (1254 of 25 966 patients) in the week before fall DST, 4.9% (1303 or 26 539 patients) in the week of fall DST, and 4.7% (1252 of 26 717 patients) in the week after fall DST. The rates of in-hospital stroke are listed in Table 3, which describes the adjusted odds ratio of having an adverse in-hospital clinical event in the week of DST compared with the weeks before or after for all patients with AMI. There was no significant difference in outcomes between patients who arrived in the fall or spring DST week vs arrived 1 week before, or vs patients who arrived 1 week after the DST week. eTables 6 and 7 in Supplement 1 shows the adjusted odds ratio of adverse clinical events for patients with STEMI and NSTEMI, respectively, comparing the week of DST with the weeks before or after DST. There was no significant difference in outcomes between patients who arrived in the fall or spring DST week vs arrived 1 week before or after.

**Table 3.** Adjusted Odds Ratios (aORs) of Clinical Adverse Events During the Daylight Savings Time (DST) Week vs the Week Before or After DST Among All Patients Presenting With Acute Myocardial Infarction^a,b^

Outcome	aOR (95% CI)	Adjusted *P* value
**In-hospital death**		
Spring DST week vs 1 week prior	1.02 (0.93-1.12)	.63
Spring DST week vs 1 week after	1.01 (0.92-1.11)	.78
Fall DST week vs 1 week prior	1.00 (0.92-1.09)	.99
Fall DST week vs 1 week after	1.02 (0.93-1.12)	.67
**In-hospital any stroke**		
Spring DST week vs 1 week prior	0.97 (0.80-1.17)	.74
Spring DST week vs 1 week after	1.14 (0.93-1.38)	.20
Fall DST week vs 1 week prior	0.95 (0.78-1.15)	.58
Fall DST week vs 1 week after	0.98 (0.82-1.18)	.84

^a^ Covariates for risk adjustment included: age, sex, race, body mass index, heart failure on presentation, cardiogenic shock on presentation, heart rate (beats per minute) and systolic blood pressure (mmHg) on presentation, cardiac arrest, presentation for ST-elevation myocardial infarction (STEMI) vs non-STEMI, prior myocardial infarction, prior percutaneous coronary intervention, prior coronary artery bypass grafting, diabetes, prior heart failure, dialysis status, peripheral artery disease, prior cardiovascular disease, dyslipidemia, hypertension, left ventricular ejection fraction (%), initial estimated glomerular filtration rate, initial troponin (upper level of normal [ULN]), and admitted year before 2020 vs after 2020.

^b^ Overall spring DST population: 81 140; overall fall DST population: 79 222.

### Sensitivity Analyses

We examined the incidence ratios for AMI, comparing the incidence during the DST weeks with the weeks before or after the DST week in the states of Hawaii and Arizona, where DST does not exist. eTable 8 in Supplement 1 depicts the IR for the whole study period by spring vs fall DST and by AMI type. The IR for these 2 states were similar over the study period, regardless of AMI type.

eFigure 3 in Supplement 1 describes the IRs for DST weeks vs 3 weeks before or 3 weeks after the DST week for the full study period. Similar to eFigure 2, there was an increase in the IR of AMI in the 3 weeks after spring DST during the year 2020. There were no other significant differences between the IRs 3 weeks before or after the DST weeks.

Excluding the 27 235 patients who presented during the first year of the COVID-19 pandemic (ie, 2020 to 2021), we examined the IRs, comparing the DST weeks vs 1 week before or after the DST week. eTable 9 in Supplement 1 lists the IR (95% CI) for each of these comparisons. The trends were similar when excluding 2020 to 2021 when compared with the entire study period.

## Discussion

To our knowledge, this is the largest analysis to date of the association of incidence of AMI and DST. Our findings showed that there was no significant increase in incidence of AMI during the week of DST or in the weeks thereafter. Additionally, we demonstrated that the adjusted risk for in-hospital death, stroke, or reperfusion (STEMI) or revascularization (NSTEMI) was not significantly different 1 week before or after the week of spring or fall DST time changes. Only in the week after spring DST in 2020 was there a marked increase in AMI incidence, a time which would have overlapped with the beginning of the COVID-19 pandemic. These findings contrast to previous smaller studies which have demonstrated a rise in the incidence of AMI in the week after spring DST.

Sleep deprivation and poor sleep hygiene have long been associated with an increased risk of AMI. Observational data from more than 450 000 participants in the UK Biobank suggested that extremes of sleep duration (less than 6 hours and more than 9 hours) were associated with an increased risk of AMI.^5^ For those participants who slept for short durations of time, 1 hour extra of sleep conferred a 20% reduction in AMI. In the prospective Stockholm Heart Epidemiology Program (SHEEP), women who had consistent disturbed sleep had a higher risk of long-term cardiovascular events: AMI (hazard ratio [HR], 1.69; 95% CI, 0.95-3.00), stroke (HR, 2.61; 95% CI, 1.19-5.76), and heart failure (HR, 2.43; 95% CI, 1.18-4.97).^6^ Previous smaller analyses have demonstrated an association between increased incidence in AMI during the days to week after spring DST,^1,2,3^ when patients may be experiencing a sudden shift in sleep schedule with a loss of an hour of sleep. However, our analysis of nearly 170 000 patients with AMI over nearly a decade study window did not demonstrate any association between increased incidence of AMI and changes in sleeping hours that occur during DST weeks.

There are multiple explanations for the different results in the current analysis from previous studies. The size of our study cohort is much larger and more heterogenous (broad US population) compared with prior studies.^1,2,3^ In fact, a recently published analysis reported that the finding of an association between an increased risk of AMI and DST found in many previous DST studies were underpowered to adequately examine the AMI risk.^7^ Another analysis cautioned against interpreting the modest increase in AMI found to be associated with DST, and recommended examining places were DST is not practiced, which our current analysis accomplished by analyzing Hawaii and Arizona.^8^ There may also be differing adjustments to DST between the US and other countries, based on work schedules and transitions to work from home schedules after the COVID-19 pandemic, which may make the DST transitions less difficult for workers and for circadian rhythms. It may also be that the significant improvements in secondary medical therapy for the post-AMI patient have improved over the past decade to the extent that the modest differences in post-AMI outcomes seen in smaller DST studies are not statistically significant in our study. Increasingly, as society has become more connected digitally with fewer work-hour boundaries, a 1-hour change in daylight hours may not be associated with a large change in clinical outcomes.

While we did not demonstrate an association between the time changes that occur during DST and AMI incidence as in previous studies, there are many disease states and clinical outcomes that have been demonstrated to be associated with the time changes that result from DST. For example, the risk of out of hospital cardiac arrest (OHCA) has been shown to be significantly increased by 13% in the days immediately following spring DST (when an hour of sleep is lost) vs a 12% reduced risk of OHCA immediately following fall DST.^9^ Additionally, DST transitions have been associated with an increased risk of ischemic stroke in the days after DST transition.^10^ While DST may be associated with various have health effects, it has important associations with human behavior. For instance, there is a significant rise in vehicular crashes in the week after DST.^11^ More recently, lawmakers have debated whether DST should be discontinued as a practice in the United States.

### Limitations

There are several important limitations. First, with any retrospective, observational study, there may be residual confounding and/or information bias present. DST may have resulted in delayed transport or care. The patterns present during DST in 2020 may be associated with the ongoing COVID-19 pandemic, as this year differed from other years during the study window. There are many reasons that the incidence of AMI may rise during different time periods (ie, a surge in incidence of flu and other respiratory viruses), so the incidence of AMI during these time periods examined in the study may not just be associated with DST.

## Conclusions

Our contemporary analysis of the association between AMI and DST revealed no significant difference in the IRs of AMI in DST weeks compared with the week before or after. Additionally, there were no differences in in-hospital clinical outcomes.

## Notes

Supplement 1. **eFigure 1. **Consort Diagram of Study Population and Cohort Exclusions **eFigure 2. **Incidence Ratios (IRs) of Acute Myocardial Infarction Presentations for Each Day of the Week of Spring Daylight Savings Time (DST) and Fall DST **eFigure 3.** Incidence Ratios (IRs) for Daylight Savings Time (DST) Weeks vs 3 Weeks After the DST Week for the Full Study Period **eTable 1.** Spring and Fall DST Weeks **eTable 2.** Covariates for Adjustment for the Multivariable Regression Model of the Association of DST With the Odds of In-Hospital Events **eTable 3. **Incidence Ratios (IRs) of AMI for a Daylight Savings Time (DST) Week vs the Week Before or After a DST Week **eTable 4.** No. (%) of In-Hospital Clinical Events by Timing Before, During, and After Spring Daylight Savings Time (DST) **eTable 5. **Number (Percentage) of In-Hospital Clinical Events by Timing Before, During and After Fall DST **eTable 6. **Adjusted Odds Ratios of Clinical Adverse Events During the DST Week vs the Week Before or After DST among Patients Presenting With STEMI **eTable 7.** Adjusted Odds Ratios of Clinical Adverse Events During the DST Week vs the Week Before or After DST Among Patients Presenting With NSTEMI **eTable 8.** Incidence Ratios (95% CIs) of AMI for a DST Week vs the Week Before or After a DST Week in Patients Presenting With AMI in Arizona and Hawaii **eTable 9.** Incidence Ratios (95% CIs) of AMI for a DST Week vs the Week Before or After a DST Week When Excluding Patients Who Presented Between 2020 to 2021

Supplement 2.
Data Sharing Statement
