# Supplementary material for: Daylight Savings Time and Acute Myocardial Infarction
Source: JAMA Netw Open. 2025 Sep 9;8(9):e2530442. doi: 10.1001/jamanetworkopen.2025.30442 (PMC12421335; doi:10.1001/jamanetworkopen.2025.30442)
Supplement: Supplement 1. — eFigure 1. Consort Diagram of Study Population and Cohort Exclusions eFigure 2. Incidence Ratios (IRs) of Acute Myocardial Infarction Presentations for Each Day of the Week of Spring Daylight Savings Time (DST) and Fall DST eFigure 3. Incidence Ratios (IRs) for Daylight Savings Time (DST) Weeks vs 3 Weeks After the DST Week for the Full Study Period eTable 1. Spring and Fall DST Weeks eTable 2. Covariates for Adjustment for the Multivariable Regression Model of the Association of DST With the Odds of In-Hospital Events eTable 3. Incidence Ratios (IRs) of AMI for a Daylight Savings Time (DST) Week vs the Week Before or After a DST Week eTable 4. No. (%) of In-Hospital Clinical Events by Timing Before, During, and After Spring Daylight Savings Time (DST) eTable 5. Number (Percentage) of In-Hospital Clinical Events by Timing Before, During and After Fall DST eTable 6. Adjusted Odds Ratios of Clinical Adverse Events During the DST Week vs the Week Before or After DST among Patients Presenting With STEMI eTable 7. Adjusted Odds Ratios of Clinical Adverse Events During the DST Week vs the Week Before or After DST Among Patients Presenting With NSTEMI eTable 8. Incidence Ratios (95% CIs) of AMI for a DST Week vs the Week Before or After a DST Week in Patients Presenting With AMI in Arizona and Hawaii eTable 9. Incidence Ratios (95% CIs) of AMI for a DST Week vs the Week Before or After a DST Week When Excluding Patients Who Presented Between 2020 to 2021 [file jamanetwopen-e2530442-s001.pdf]

Supplemental Online Content

Rymer JA, Li S, Chiswell K, et al. Daylight savings time and acute myocardial infarction. *JAMA Network Open.* 2025;8(9):e2530442.
doi:10.1001/jamanetworkopen.2025.30442

eFigure 1. Consort Diagram of Study Population and Cohort Exclusions

eFigure 2. Incidence Ratios (IRs) of Acute Myocardial Infarction Presentations for Each Day of the Week of Spring Daylight Savings Time (DST) and Fall DST

eFigure 3. Incidence Ratios (IRs) for Daylight Savings Time (DST) Weeks vs 3 Weeks After the DST Week for the Full Study Period

eTable 1. Spring and Fall DST Weeks

eTable 2. Covariates for Adjustment for the Multivariable Regression Model of the Association of DST With the Odds of In-Hospital Events

eTable 3. Incidence Ratios (IRs) of AMI for a Daylight Savings Time (DST) Week vs the Week Before or After a DST Week

eTable 4. No. (%) of In-Hospital Clinical Events by Timing Before, During, and After Spring Daylight Savings Time (DST)

eTable 5. Number (Percentage) of In-Hospital Clinical Events by Timing Before, During and After Fall DST

eTable 6. Adjusted Odds Ratios of Clinical Adverse Events During the DST Week vs the Week Before or After DST among Patients Presenting With STEMI

eTable 7. Adjusted Odds Ratios of Clinical Adverse Events During the DST Week vs the Week Before or After DST Among Patients Presenting With NSTEMI

eTable 8. Incidence Ratios (95% CIs) of MI for a DST Week vs the Week Before or After a DST Week in Patients Presenting With AMI in Arizona and Hawaii

eTable 9. Incidence Ratios (95% CIs) of AMI for a DST Week vs the Week Before or After a DST Week When Excluding Patients Who Presented Between 2020 to 2021

This supplemental material has been provided by the authors to give readers additional information about their work.

© 2025 Rymer JA et al. *JAMA Network Open.*

eFigure 1. Consort Diagram of Study Population and Cohort Exclusions. The beginning study cohort and excluded patients are described along with the final study population.

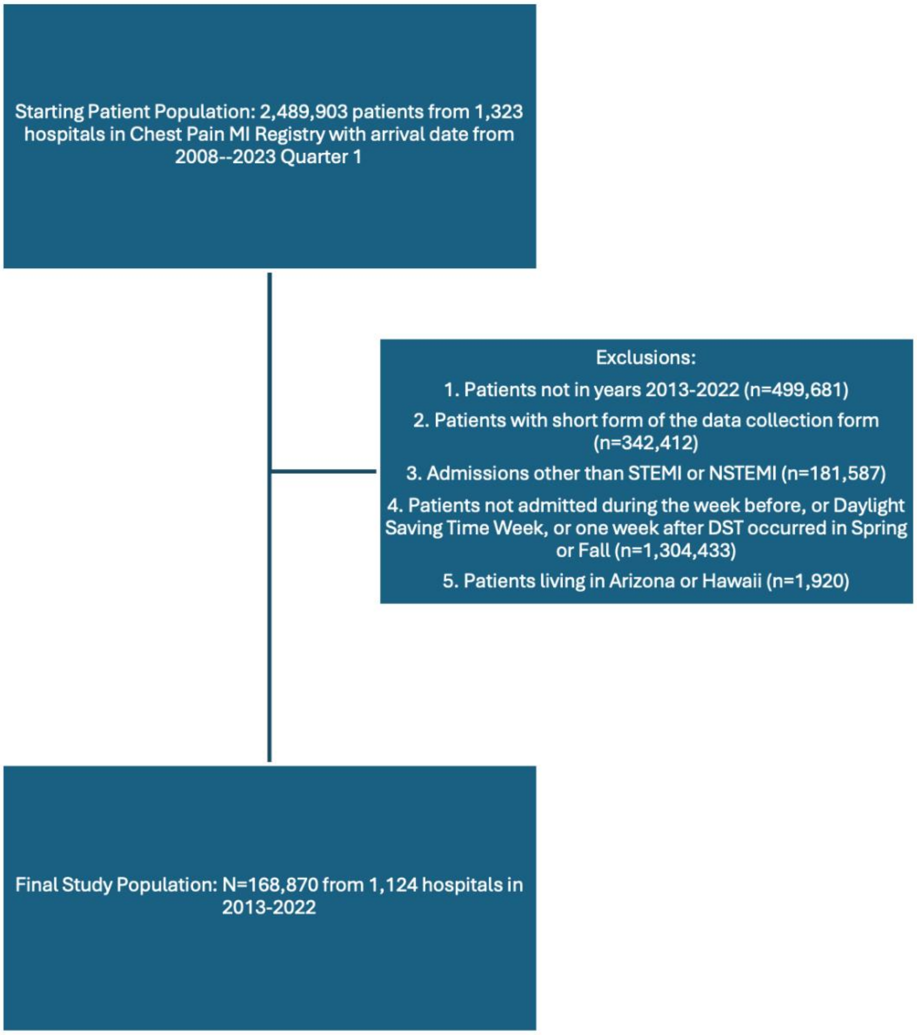

© 2025 Rymer JA et al. *JAMA Network Open*.

eFigure 2. Incidence Ratios (IRs) of Acute Myocardial Infarction Presentations for Each Day of the Week of Spring Daylight Savings Time (DST) and Fall DST

Incidence ratios (IR) of AMI presentations for each day of the week of Spring DST (**1A**) and Fall DST (**1B**) versus the week prior, and versus the week after for the entire study period. By day of the week, there was no significant difference between the IRs of AMI presentations for the week of Spring DST and Fall DST versus the week prior, and versus the week after for the entire study period.

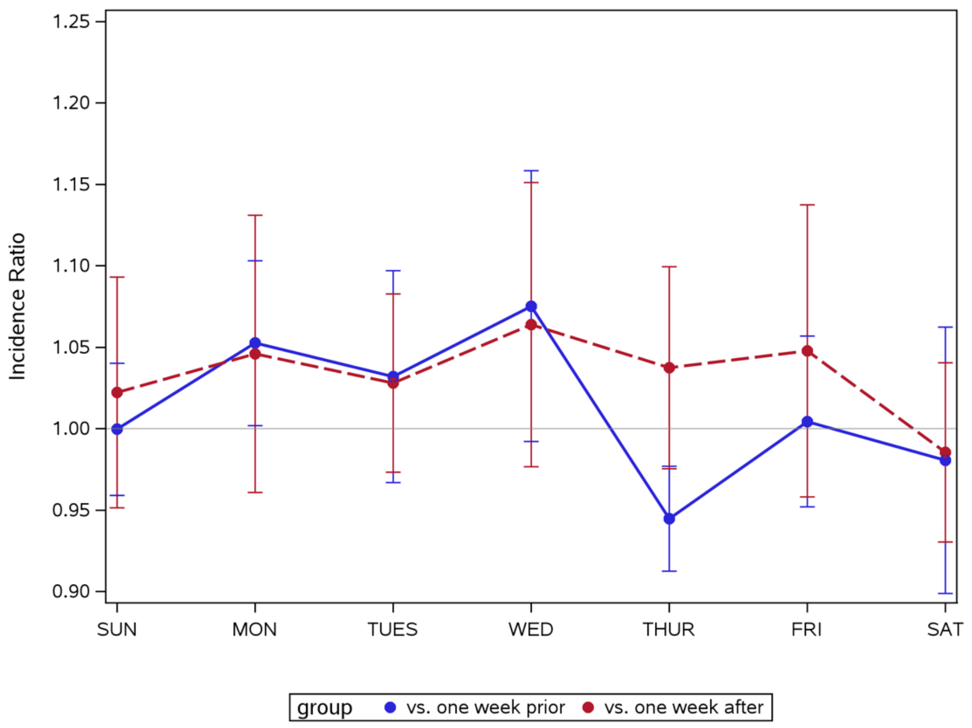

© 2025 Rymer JA et al. *JAMA Network Open*.

eFigure 2B.

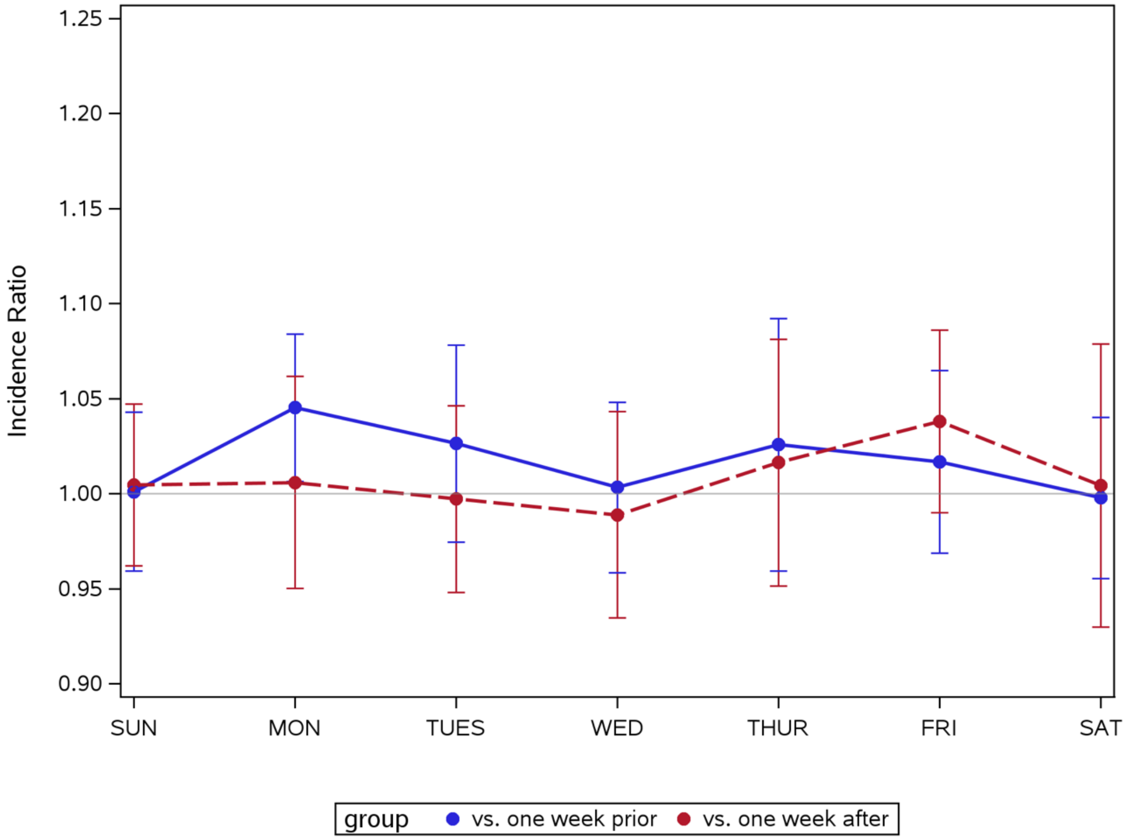

© 2025 Rymer JA et al. *JAMA Network Open*.

eFigure 3. Incidence Ratios (IRs) for Daylight Savings Time (DST) Weeks vs 3 Weeks After the DST Week for the Full Study Period

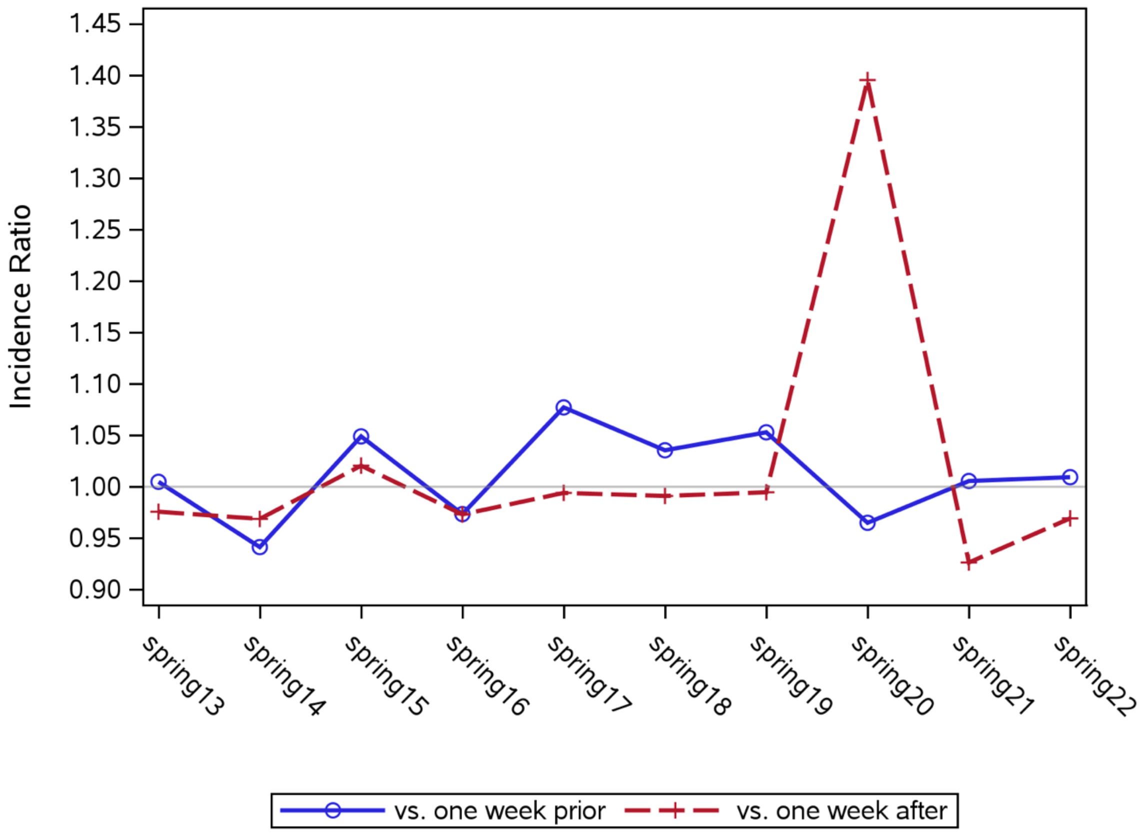

© 2025 Rymer JA et al. *JAMA Network Open*.

eFigure 3B.

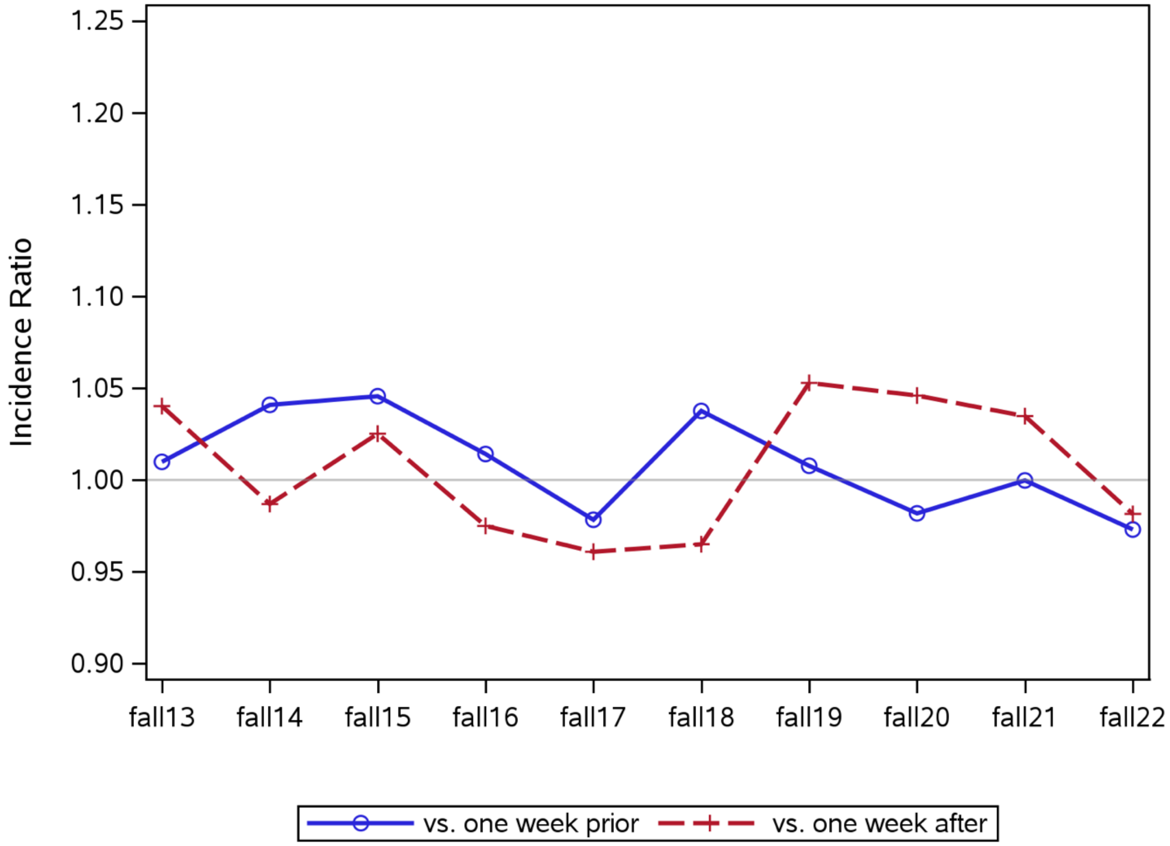

© 2025 Rymer JA et al. *JAMA Network Open*.

eTable 1. Spring and Fall DST Weeks

Year	Spring		
	One week Before	DST Week	One Week After
2013	March 3-9	March 10-16	March 17-23
2014	March 2-8	March 9-15	March 16-22
2015	March 1-7	March 8-14	March 15-21
2016	March 6-12	March 13-19	March 20-26
2017	March 5-11	March 12-18	March 19-25
2018	March 4-10	March 11-17	March 18-24
2019	March 3-9	March 10-16	March 17-23
2020	March 1-7	March 8-14	March 15-21
2021	March 7-13	March 14-20	March 21-27
2022	March 6-12	March 13-19	March 20-26

Year	Fall		
	One week Before	DST Week	One Week After
2013	October 27-November 2	November 3-9	November 10-16
2014	October 26-November 1	November 2-8	November 9-15
2015	October 25-October 31	November 1-7	November 8-14
2016	October 30-November 5	November 6-12	November 13-19
2017	October 29-November 4	November 5-11	November 12-18
2018	October 28-November 3	November 4-10	November 11-17
2019	October 27-November 2	November 3-9	November 10-16
2020	October 25-October 31	November 1-7	November 8-14
2021	October 31-November 6	November 7-13	November 14-20
2022	October 30-November 5	November 6-12	November 13-19

© 2025 Rymer JA et al. *JAMA Network Open*.

eTable 2. Covariates for Adjustment for the multivariable regression model of the association of DST with the odds of in-hospital events

**Covariates for Adjustment**	Covariates for risk adjustment included: age, sex, race, BMI (kg/m^2), heart failure on presentation, cardiogenic shock on presentation, heart rate (bpm) and systolic blood pressure (mmHg) on presentation, cardiac arrest, presentation for STEMI vs. NSTEMI, prior MI, prior PCI, prior CABG, diabetes, prior heart failure, dialysis status, peripheral artery disease, prior CVD, dyslipidemia, hypertension, left ventricular ejection fraction (%), initial estimated glomerular filtration rate, initial troponin (xULN), and admitted year before 2020 vs. after 2020

© 2025 Rymer JA et al. *JAMA Network Open*.

eTable 3. Incidence Ratios (IRs) of AMI for a Daylight Savings Time (DST) Week vs the Week Before or After a DST Week

(Spring STEMI DST Population: 32,087; Fall STEMI DST Population: 31,368; Spring NSTEMI DST Population: 53,356; Fall NSTEMI DST Population: 52,059)

	AMI Overall	STEMI	NSTEMI
**Spring: DST week versus one week prior**	1.01 (0.99 – 1.03)	1.01 (0.98 – 1.05)	1.01 (0.98 – 1.03)
**Spring: DST week versus one week after**	1.03 (0.98 – 1.08)	1.02 (0.96 – 1.08)	1.04 (0.98 – 1.09)
**Fall: DST week versus one week prior**	1.02 (0.99 – 1.04)	1.03 (0.99 – 1.07)	1.01 (0.98 – 1.04)
**Fall: DST week versus one week after**	1.00 (0.98 – 1.02)	1.01 (0.97 – 1.05)	1.00 (0.97 – 1.02)

AMI = acute myocardial infarction, STEMI = ST elevation myocardial infarction, NSTEMI = non-ST elevation myocardial infarction

© 2025 Rymer JA et al. *JAMA Network Open*.

eTable 4. No. (%) of In-Hospital Clinical Events by Timing Before, During, and After Spring Daylight Savings Time (DST)

	Overall 81140 (%)	One Week before Spring DST week 27152 (%)	Spring DST Week 27235 (%)	One Week after Spring DST week 26753 (%)
**In-Hospital Clinical Events**				
In-Hospital Death	3,651 (4.5)	1,223 (4.5)	1,245 (4.6)	1,183 (4.4)
In-Hospital Stroke	646 (0.8)	230 (0.9)	224 (0.8)	192 (0.7)

© 2025 Rymer JA et al. *JAMA Network Open*.

eTable 5. Number (Percentage) of In-Hospital Clinical Events by Timing Before, During and After Fall DST

	Overall 79222 (%)	One Week before Fall DST week 25966 (%)	Fall DST Week 26539 (%)	One Week after Fall DST week 26717 (%)
**In-Hospital Clinical Events**				
In-Hospital Death	3,809 (4.8)	1,254 (4.8)	1,303 (4.9)	1,252 (4.7)
In-Hospital Stroke	651 (0.8)	220 (0.9)	213 (0.8)	218 (0.8)

© 2025 Rymer JA et al. *JAMA Network Open*.

eTable 6. Adjusted Odds Ratios of Clinical Adverse Events during the DST Week vs. the Week Before or After DST among Patients Presenting with STEMI (Spring STEMI DST Population: 32,087; Fall STEMI DST Population: 31,368)

Outcome	Group	Adjusted OR (95% CI)	Adjusted p-value
In-hospital Death	Spring DST week vs. one week prior	1.06 (0.94 - 1.21)	0.35
	Spring DST week vs. one week after	0.98 (0.86 - 1.11)	0.72
	Fall DST week vs. one week prior	1.03 (0.91 - 1.17)	0.64
	Fall DST week vs. one week after	0.98 (0.87 - 1.11)	0.75
In-hospital any Stroke	Spring DST week vs. one week prior	1.02 (0.78 - 1.35)	0.87
	Spring DST week vs. one week after	1.34 (0.99 - 1.81)	0.06
	Fall DST week vs. one week prior	0.90 (0.69 - 1.18)	0.46
	Fall DST week vs. one week after	0.97 (0.74 - 1.26)	0.80
Reperfusion for STEMI	Spring DST week vs. one week prior	1.06 (0.97 - 1.17)	0.21
	Spring DST week vs. one week after	1.03 (0.94 - 1.14)	0.47
	Fall DST week vs. one week prior	1.04 (0.94 - 1.15)	0.42
	Fall DST week vs. one week after	1.01 (0.92 - 1.11)	0.86

© 2025 Rymer JA et al. *JAMA Network Open*.

eTable 7. Adjusted Odds Ratios of Clinical Adverse Events during the DST Week vs. the Week Before or After DST among Patients Presenting with NSTEMI (Spring NSTEMI DST Population: 53,356; Fall NSTEMI DST Population: 52,059)

Outcome	Group	Adjusted OR (95% CI)	Adjusted p-value
In-hospital Death	Spring DST week vs. one week prior	0.98 (0.86 - 1.11)	0.72
	Spring DST week vs. one week after	1.06 (0.92 - 1.22)	0.45
	Fall DST week vs. one week prior	0.98 (0.86 - 1.11)	0.73
	Fall DST week vs. one week after	1.06 (0.93 - 1.21)	0.37
In-hospital any Stroke	Spring DST week vs. one week prior	0.91 (0.71 - 1.15)	0.42
	Spring DST week vs. one week after	1.00 (0.78 - 1.29)	0.98
	Fall DST week vs. one week prior	0.99 (0.76 - 1.30)	0.96
	Fall DST week vs. one week after	1.00 (0.77 - 1.30)	0.98
Revascularization for NSTEMI	Spring DST week vs. one week prior	0.97 (0.93 - 1.01)	0.12
	Spring DST week vs. one week after	1.00 (0.96 - 1.05)	0.90
	Fall DST week vs. one week prior	0.97 (0.93 - 1.01)	0.10
	Fall DST week vs. one week after	0.97 (0.93 - 1.01)	0.17

NSTEMI = non-ST elevation myocardial infarction

© 2025 Rymer JA et al. *JAMA Network Open*.

eTable 8. Incidence Ratios (95% Confidence Interval) of MI for a DST Week versus the Week before or after a DST Week in Patients Presenting with AMI in Arizona and Hawaii (N=1920)

	AMI	STEMI	NSTEMI
**Spring: DST week versus one week prior**	0.85 (0.67, 1.03)	1.07 (0.67, 1.46)	0.84 (0.52, 1.15)
**Spring: DST week versus one week after**	1.01 (0.85, 1.17)	1.14 (0.88, 1.39)	0.96 (0.74, 1.18)
**Fall: DST week versus one week prior**	0.96 (0.77, 1.15)	1.13 (0.81, 1.44)	0.92 (0.64, 1.20)
**Fall: DST week versus one week after**	0.94 (0.78, 1.09)	1.27 (0.66, 1.87)	0.96 (0.64, 1.28)

AMI = acute myocardial infarction, STEMI = ST-elevation myocardial infarction, NSTEMI = non-ST elevation myocardial infarction

© 2025 Rymer JA et al. *JAMA Network Open*.

eTable 9. Incidence Ratios (95% Confidence Interval) of AMI for a DST Week versus the Week before or after a DST Week When Excluding Patients who Presented between 2020-2021 (First Year of COVID-19 Pandemic) (N=27325)

	AMI	STEMI	NSTEMI
Spring: DST week versus one week prior	1.02 (1.00, 1.04)	1.02 (0.98, 1.07)	1.01 (0.99, 1.04)
Spring: DST week versus one week after	1.01 (0.99, 1.04)	1.02 (0.98, 1.07)	1.01 (0.99, 1.03)
Fall: DST week versus one week prior	1.01 (0.98, 1.05)	1.02 (0.98, 1.07)	1.01 (0.97, 1.05)
Fall: DST week versus one week after	1.00 (0.97, 1.02)	1.00 (0.95, 1.04)	1.00 (0.97, 1.03)

© 2025 Rymer JA et al. *JAMA Network Open*.
