# Supplementary material for: Daylight Savings Time and Acute Myocardial Infarction
Source: JAMA Netw Open. 2025 Sep 9;8(9):e2530442. doi: 10.1001/jamanetworkopen.2025.30442 (PMC12421335; doi:10.1001/jamanetworkopen.2025.30442)
Supplement: Supplement 2. — Data Sharing Statement [file jamanetwopen-e2530442-s002.pdf]

Data Sharing Statement

Data
Data available: No

Additional Information
Explanation for why data not available: All data requests would need to be made to the ACC National Cardiovascular Data Registry.
